# Lesion Induced Error on Automated Measures of Brain Volume: Data From a Pediatric Traumatic Brain Injury Cohort

**DOI:** 10.3389/fnins.2020.491478

**Published:** 2020-11-30

**Authors:** Daniel J. King, Jan Novak, Adam J. Shephard, Richard Beare, Vicki A. Anderson, Amanda G. Wood

**Affiliations:** ^1^College of Health and Life Sciences, Aston Institute of Health and Neurodevelopment, Aston University, Birmingham, United Kingdom; ^2^Developmental Imaging, Clinical Sciences, Murdoch Children's Research Institute, Melbourne, VIC, Australia; ^3^Medicine, Peninsula Clinical School, Monash University, Melbourne, VIC, Australia; ^4^Brain and Mind Research, Clinical Sciences, Murdoch Children's Research Institute, Melbourne, VIC, Australia; ^5^Department of Psychology, Royal Children's Hospital, Melbourne, VIC, Australia; ^6^Faculty of Health, School of Psychology, Deakin University Melbourne Burwood Campus, Geelong, VIC, Australia

**Keywords:** traumatic brain injury, lesion, FreeSurfer, morphometry, pediatric, methods

## Abstract

Structural segmentation of T1-weighted (T1w) MRI has shown morphometric differences, both compared to controls and longitudinally, following a traumatic brain injury (TBI). While many patients with TBI present with abnormalities on structural MRI images, most neuroimaging software packages have not been systematically evaluated for accuracy in the presence of these pathology-related MRI abnormalities. The current study aimed to assess whether acute MRI lesions (MRI acquired 7–71 days post-injury) cause error in the estimates of brain volume produced by the semi-automated segmentation tool, Freesurfer. More specifically, to investigate whether this error was global, the presence of lesion-induced error in the contralesional hemisphere, where no abnormal signal was present, was measured. A dataset of 176 simulated lesion cases was generated using actual lesions from 16 pediatric TBI (pTBI) cases recruited from the emergency department and 11 typically-developing controls. Simulated lesion cases were compared to the “ground truth” of the non-lesion control-case T1w images. Using linear mixed-effects models, results showed that hemispheric measures of cortex volume were significantly lower in the contralesional-hemisphere compared to the ground truth. Interestingly, however, cortex volume (and cerebral white matter volume) were not significantly different in the lesioned hemisphere. However, percent volume difference (PVD) between the simulated lesion and ground truth showed that the magnitude of difference of cortex volume in the contralesional-hemisphere (mean PVD = 0.37%) was significantly smaller than that in the lesioned hemisphere (mean PVD = 0.47%), suggesting a small, but systematic lesion-induced error. Lesion characteristics that could explain variance in the PVD for each hemisphere were investigated. Taken together, these results suggest that the lesion-induced error caused by simulated lesions was not focal, but globally distributed. Previous post-processing approaches to adjust for lesions in structural analyses address the focal region where the lesion was located however, our results suggest that focal correction approaches are insufficient for the global error in morphometric measures of the injured brain.

## Introduction

Automated analysis to derive quantitative measures of brain structure offers significant benefit to large scale research endeavors that have clinical translation potential. In addition to reducing the time burden and potential error induced by manual methods (Bigler et al., 2010), quantitative approaches may be more sensitive to subtle but clinically-relevant imaging biomarkers that are not apparent on routine visual reporting. Accordingly, successful use of automated techniques has been demonstrated in disorders with relatively subtle global or regional changes [e.g., dementia of the Alzheimer's type (Frisoni et al., 2010)]. Recent traumatic brain injury (TBI) research has utilized segmentation and analysis of T1-weighted (T1w) structural magnetic resonance images (MRI) to quantify the post-injury morphometric changes [Dennis et al., 2017; Ryan et al., 2017; Urban et al., 2017, see King et al. (2019) for a review]. The accuracy of automated methods in the context of gross lesions/pathology, however, may be reduced by errors introduced during the processing of such MRI. This then makes it difficult to ascertain whether differences between control and patient morphology are due to an injury-related pathology or due to systematic error which is specific to the patient cases with gross lesions (King et al., 2019).

The MRI features of TBI are heterogeneous due to injury mechanisms such as white matter deformation and shear, Wallerian degeneration, compromised vasculature, hemosiderin deposits and encephalomalacia (Bigler and Wilde, 2010; Bigler et al., 2013, 2016), presenting as abnormal signal within the image, hereto referred to as “lesions.” The current study investigated MRI lesions in a cohort of pediatric TBI patients. In TBI cases, lesions occur within the context of a still-developing brain (Wilde et al., 2012a), and accurate quantification of brain-morphology will allow us to assess the effects of traumatic brain insults on the developmental trajectory of the brain.

Lesions are not uncommon in this population; in a retrospective, accidental pediatric TBI (pTBI) cohort (*n* = 68), MRI within 2 weeks detected intraparenchymal lesions on ~29% of cases (Buttram et al., 2015). In a study of 36 patients, lesions were detected on MRI (T1w, T2w, or FLAIR) for ~56% of cases [*n* = 20, Beauchamp et al. (2011)]. However, this is likely an inflated prevalence as Beauchamp et al. (2011) specifically included only those patients who explicitly had been clinically referred for CT.

pTBI lesions can be as unique between individuals as the precipitating injury, with no two individuals sharing the same biomechanics of injury, genetic context, or experience-dependent plasticity (Saatman et al., 2008). This means that the presentation of lesions on MR imaging is highly variable between individuals, but also within-cases. The pattern of pathology varies across time post-injury: for example, white-matter shear is more common acutely, whilst Wallerian degeneration is a late manifestation of injury. Even for a given individual, lesion presentation on MRI is highly dependent on factors such as MR sequence and time post-injury (Bigler, 2007; Bigler and Maxwell, 2011; Bigler et al., 2013). This heterogeneity means that lesion characterization presents a major challenge for neuroimaging software and analysis.

There are multiple potential sources of error in neuroimaging pipelines due to the presence of lesions. Frank parenchymal lesions in TBI can result in abnormal voxel intensities (due to pathology such as gliosis and oedema), distorting image-processing of intensity gradients (Merkley et al., 2008; Irimia et al., 2012; Goh et al., 2014). Gross TBI pathology can also lead to inaccurate voxel-mappings to brain-atlas space, biasing estimates of structural TBI volumetrics (Irimia et al., 2012; Goh et al., 2014). Many automated approaches to segmentation are therefore likely to show a lesion-induced error when used to process MRI from clinical populations with visible lesions and pathology.

Regardless of their exact origin within the processing pipeline, the effect of errors in the automated processing of these MRI is the potential to obscure or falsely identify findings of pathology-mediated changes to the morphology of the brain. For example, focal white matter (WM) lesions seen in multiple sclerosis have been shown to bias measures derived from SPM (Penny et al., 2011), FIRST (Patenaude et al., 2011), Freesurfer (Fischl, 2012), and multi-atlas segmentation methods (Chard et al., 2010; Gonzalez-Villa et al., 2017).

Freesurfer (Fischl, 2012) is a tool for the semi-automated segmentation of T1w structural MRI to estimate the morphometry of the brain. Whilst other structural neuroimaging pipelines are available, Freesurfer utilizes a 2D surface based approach which holds advantages over 3D volume based approaches, including better adherence to the cortical geometry (Fischl, 2012; Dickie et al., 2019). Documentation does not discuss how to approach surface-based segmentation of lesioned images but does emphasize that the tool should not be used for clinical purposes. To date, little work has investigated Freesurfer's performance in the presence of pathology-related MRI abnormalities. Despite these limitations, it has been disproportionately used in pTBI studies [e.g., Mayer et al. (2015); Drijkoningen et al. (2017); Ryan et al. (2017); Wilde et al. (2012b); Wu et al. (2018)]. The majority of the pTBI studies listed here report little detail on the implementation of Freesurfer in the presence of TBI-lesions, beyond the fact that manual-editing was performed. This paucity of detail restricts the ability to replicate study findings and assess the effect lesions may have on the Freesurfer pipeline.

Despite a lack of research into methodologies to approach automated segmentation in the presence of lesions, previous studies investigating brain morphometry in pTBI have reported and adopted strategies to deal with the effect of lesions on their analyses. One utilized approach is to exclude cases with focal lesions from analyses (Serra-Grabulosa et al., 2005), however, this both reduces statistical power (through reduced sample size) and limits clinical applicability and generalizability of findings to the full spectrum of injuries. Other studies have used *post-hoc* procedures to “correct” for the effect of lesions on their analyses by replicating analyses with/without patients presenting with focal lesions in the region of interest (ROI) being tested (Spanos et al., 2007), or excluding ROIs where the presence of a lesion caused errors to the Freesurfer parcellation (Drijkoningen et al., 2017). The *post-hoc* correction methods outlined here rely on the assumption that the lesion-induced error is focal. However, it is important to consider whether this algorithmic error could be distributed globally across the brain, causing an error in regions not edited by these correction approaches.

We aimed to identify and quantify this potential global lesion-induced error by simulating TBI-lesions in a healthy pediatric cohort. Simulated lesions facilitate measurement of the effect of image processing in the presence of a lesion as compared to the “ground truth,” the non-lesioned counterpart of the image. This is necessary to disentangle both the morphological changes due to algorithmic error and potentially “real,” globally-distributed pathological effects of the injury. To achieve our aim, we investigated morphometry in both the lesioned and contralesional hemispheres. This was to disentangle the volumetric differences due to injury and those due to the algorithmic error within the surface-based output of Freesurfer, likely induced by the erroneous signal within the lesion (Chard et al., 2010). Specifically, investigating volumetric differences in the contralesional hemisphere is the purest test of the hypothesized algorithm-induced error as, in this hemisphere, the anatomy is identical be was anatomically identical across ground-truth MRI and simulated lesion cases.

We predicted that the presence of lesions would result in an error in morphometric measurement by Freesurfer, beyond that of the spatial extent of the lesion. We had three explicit hypotheses (hypotheses a and b were defined a priori, whereas c was exploratory):

- that for cases where we have simulated a lesion there will be a difference in measured volume from ground truth in both the lesioned hemisphere, due to sections of the gray and white matter being replaced with lesioned tissue, but also the contralesional hemisphere due to systematic bias introduced by the lesion,
- that the magnitude of this difference will be greater in the lesioned hemisphere than the contralesional hemisphere,
- that this lesion-induced error (both in the lesioned and contralesional hemisphere) will vary as a function of lesion characteristics.

## Methods

### Participants

The data used in the current study represent a subset of an existing dataset of pediatric TBI. This dataset contains a total of 157 children (patients *n* = 114) who were recruited between 2007 and 2010 into a study on “Prevention and Treatment of Social Problems Following TBI in Children and Adolescents.” Further details of the study including details of the recruitment strategy have recently been published elsewhere (Anderson et al., 2013, 2017; Catroppa et al., 2017). In brief, children with TBI were recruited on presentation to The Royal Children's' Hospital, Melbourne (RCHM), Australia. Children were eligible for the study if on presentation they: (i) were aged between five and 16 years at the time of injury, (ii) had recorded evidence of both a closed-head injury and at least two post-concussive symptoms (such as headaches, dizziness, nausea, irritability, poor concentration), (iii) had sufficient detail within medical records [Glasgow Coma Scale [GCS; Teasdale and Jennett (1974)], neurological and radiological findings] with which to determine the severity of the injury, (iv) had no prior history of neurological or neurodevelopmental disorder, non-accidental injuries or previous TBI, and (v) were English speaking. TD controls were required to meet criteria (i), (iv), and (v). Of the participants recruited, 107 survivors of TBI and 36 typically developing (TD) controls had MRI scans acquired. MRI scans were acquired within the first 90 days post-injury (for details, refer to Table 1). A favorable ethical opinion was granted from Aston University as a site for secondary analysis of neuroimaging data.

**Table 1:** Injury variables for lesion cases.

**Case no**.	**Injury-MRI interval (days)**	**Injury severity**	**Cause of injury**	**Lesioned hemisphere**	**Lesion volume (mm^**3**^)**
1	35	Moderate	MVA	lh	25.00
2	40	Moderate	MVA	lh	1,030.00
3	19	Moderate	MVA	rh	3,063.75
4	35	Moderate	MVA	lh	505.75
5	7	Moderate	MVA	lh	12,081.50
6	42	Mild-complex	Fall	lh	60.25
7	29	Mild-complex	Fall	rh	63.00
8	57	Severe	Fall	rh	35.00
9	32	Mild-complex	Fall	rh	2,059.25
10	71	Moderate	Fall	rh	8,815.00
11	38	Moderate	MVA	lh	83.50
12	35	Severe	Fall	rh	3,858.50
13	20	Moderate	Fall	rh	391.50
14	36	Moderate	Fall	lh	15.00
15	43	Moderate	Fall	rh	37.50
16	63	Moderate	MVA	rh	407.00

Control cases were selected from the overall dataset based on four criteria, to ensure that the control data used was of high quality: (i) MRI data available, (ii) no manual-editing of surfaces required after Freesurfer recon-all pipeline completed, (iii) no MR-artifacts, and iv) no “failed” ratings (a “bad” rating on any of “image sharpness,” “ringing,” “subcortical SNR,” or “GM + WM SNR” scales) on a qualitative rating scale of T1w images [Backhausen et al. (2016); performed by DJK]. Eleven (out of 36) control cases met these criteria for inclusion, consisting of 5 females and 6 males, with a mean age of 9.7 yrs (range = 6.8–14.6 yrs.).

**Figure 1:**
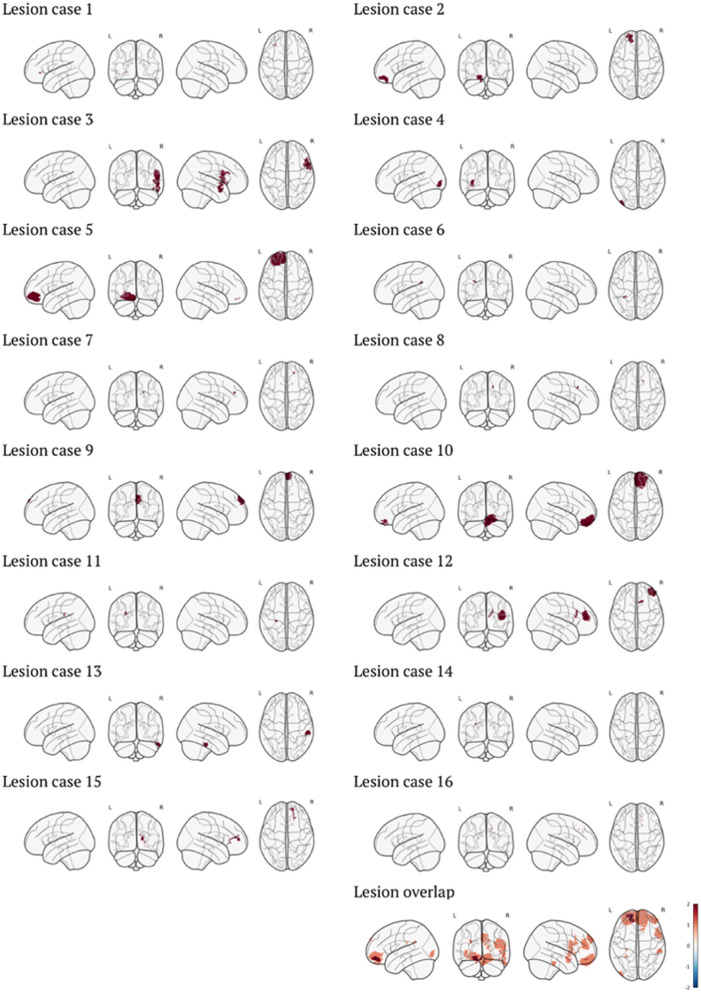
Visualization of individual lesion masks in MNI space, as well as overlap of all lesions used in the cohort (color bar represents the number of cases). Visualization generated with code from Whitaker et al. (2017).

Patients were all investigated for visible lesions on T1w images, blind to severity ratings. Nineteen patient cases were identified as presenting with lesions that could be identified on these T1w images. However, three patients were excluded from this selection. The first was excluded due to the presence of bilateral lesions which therefore precludes comparison between the lesion and contralesional hemispheres. The second exclusion was due to the resolution of the T1w image being significantly different from the other images (0.8 mm Isotropic). The final exclusion was due to the lesion being an incidental finding, rather than due to the TBI. The final sample consisted of 16 lesion cases (8/8 M/F). Mean age at injury for patients was distributed across childhood (age at injury: mean = 9.8 yrs., range = 5.8–13.7 yrs.), with MRI conducted shortly after (age at MRI: mean = 10.0 yrs., range = 5.9–13.8). Additional demographic characteristics of the lesion cases can be seen in Table 1. The included lesions were visualized as binary masks in MNI space in Figure 1. Lesion volumes (mm^3^) were calculated as a count of the number of non-zero voxels in the associated lesion mask multiplied by the voxel size. This was calculated in the native space of the lesion patient space (reported in Table 1) and once transformed into control space as a simulated lesion (see below).

### MRI Acquisition

MRIs were acquired for the patient group acutely after injury (<90 days post-injury). MRI images were acquired at 3T as a part of an existing research protocol on a Siemens Trio scanner (Siemens Medical Systems, Erlangen, Germany) using a 32-channel matrix head coil. The acquisition sequence consisted of a sagittal three-dimensional (3D) MPRAGE [TR = 1,900 ms; TE = 2.15 ms; IR prep = 900 ms; parallel imaging factor (GRAPPA) 2; flip angle 9 degrees; BW 200 Hz/Px; 176 slices; resolution 1 × 0.5 × 0.5 mm], sagittal 3D T2-w non-selective inversion preparation SPACE (Sampling Perfection with Application-optimized Contrast using different flip-angle Evolution) [TR = 6,000 ms; TE = 405 ms; inversion time (TI) = 2,100 ms; water excitation; GRAPPA Pat2; 176 slices; 1 × 0.5 × 0.5 mm resolution matched in alignment to the 3D T1w sequence].

### Simulated Lesions

All MRI processing was conducted on a Linux system (UBUNTU 16.04.4 LTS). The lesions described above were initially segmented manually (by JN with 8 years previous experience of lesion delineation in pediatric neurooncology and TBI research) using the *MRTrix (version 3.0)* software package (Tournier et al., 2012), producing a binary lesion mask for each patient. Lesions were identified in our dataset by a single rater and masks drawn where visible lesions could be identified by eye on the T1w image, using FLAIR MRI to support lesion identification.

**Figure 2:**
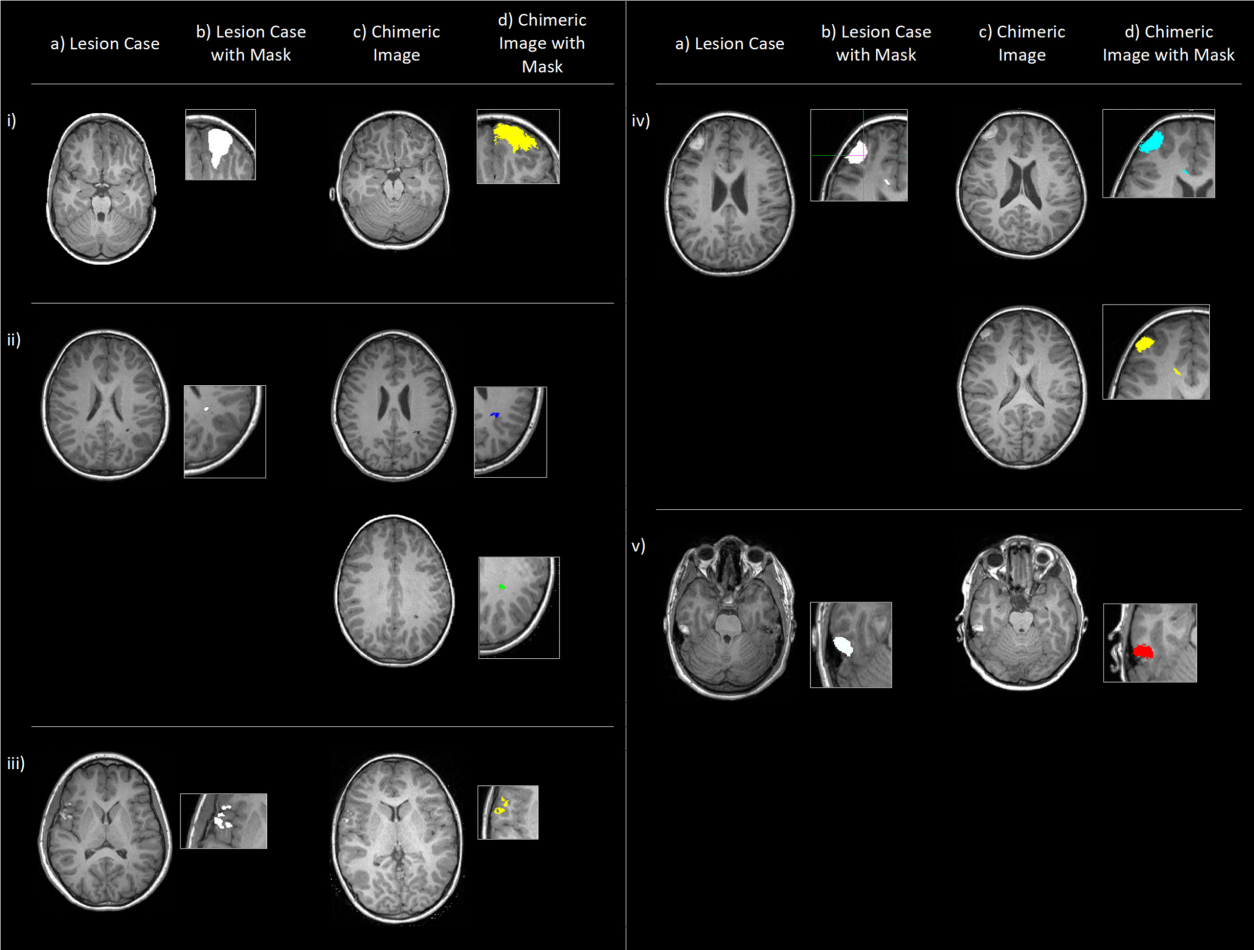
Examples (i-v) of original lesion cases **(a)**, also shown with lesion masks **(b)**, and example chimeric images **(c)**, the simulated cases where the corresponding lesion has been applied to a control subject (in native space) with the outlined methodology. **(d)** shows these chimeric images with transformed lesion masks overlaid. Cases (ii) and (iv) show two example chimeric images to demonstrate that (in native space) the simulated lesions show both morphological and spatial variation.

The approach in the current paper was similar to that proposed by Brett et al. (2001), and Gonzalez-Villa et al. (2017) using lesions from pTBI cases recruited on admission to an emergency department. The use of actual lesions provides distinct benefits over computer-generated lesions, specifically reflecting the complexity of actual lesions, retaining natural characteristics such as texture and size (Seghier et al. 2008). The full methodology is outlined in the supplementary materials and is visualized diagrammatically in Supplementary Figure 1. The resultant simulated dataset contained *n* = 176 cases, where every included lesion (*n* = 16) had been applied to every control image (*n* = 11) in all possible pairwise permutations. From here on, the control images with the simulated lesions applied will be referred to as the simulated lesion cases (*n* = 176) and the control images without editing will be referred to as “ground truth” cases (*n* = 11). Examples of these lesion images and simulated cases can be seen in Figure 2.

### Automated Structural Segmentation Using Freesurfer

Both the simulated-lesion and the ground-truth cases were processed using the standard Freesurfer recon-all pipeline (v5.3), which has good replicability and has been histologically validated (Rosas et al., 2002; Han et al., 2006). Explanations of the Freesurfer pipeline for 3D tissue segmentation and measurement of morphometry are given elsewhere (Fischl et al., 2004; Fischl, 2012). No additional “optional” processing flags were used beyond the required arguments. No manual editing was performed on either the simulated-lesion or control cases once segmented using Freesurfer to prevent any potential bias toward manual delineation (Perlaki et al., 2017).

Raw data were extracted using Freesurfer for both the simulated-lesion and ground truth cases of two volumetric measures; (a) Cortex volume [left (*lh*) and right (*rh*) hemisphere] and (b) Cerebral white matter (cWM) volume (*lh* and *rh*).

### Statistical Analysis

For each lesion applied to the control cases to generate the simulated-lesion cases, lesion masks were used to ascertain laterality of lesions. Using this information, and knowing which lesion is applied to each of the simulated-lesion cases, the raw volumetric measures for each case were recoded as the lesioned hemisphere and the contralesional hemisphere. This is the case for all metrics calculated for the study. This allowed us to see if any lesion-induced error is present in the hemisphere where no volumetric differences should occur in comparison to ground-truth, as no image manipulation has occurred in the contralesional hemisphere. For each simulated-lesion case, the appropriate reference ground-truth case was matched and also recoded to maintain the mapping from *lh*/*rh* to lesioned hemisphere/contralesional hemisphere. Thus, each repeated measure “datapoint” contained a measure of both cortex and cWM volume for; (*i*) lesioned hemisphere in the simulated-lesion case, (*ii*) contralesional hemisphere in the simulated-lesion case, (*iii*) lesioned hemisphere in the reference ground-truth case and (*iv*) contralesional hemisphere in the reference ground-truth case.

All statistical analyses were conducted in R (R Core Team, 2016) using “lme4: Linear Mixed-Effects Models using “Eigen” and S4” [lme4 (Version 1.1); Bates et al. (2015)]. All analyses utilize linear mixed-effect models [using the “*lmer*” function and restricted maximum likelihood (REML) estimation] to account for the crossed random effects of both the control MRI and lesion used to construct the simulated-lesion cases (lesion used was coded as 0 for the ground-truth cases where no lesion was applied). The random effects of lesion and control MRI used were defined as crossed, rather than nested. All model constructions are outlined in the (Supplementary Table 1).

To test hypothesis (a), cortex and cWM volumes were modeled as a function of the fixed effect of case (simulated-lesion vs. ground-truth cases) and the random effects of lesion and control MRI used to construct the simulated-lesion case. As per Barr et al.'s (2013) recommendations for best practices in mixed-effect modeling, a maximal model was defined. Barr et al. suggest that random slopes are required for within-unit effects, but random intercepts are sufficient for between-unit effects. Therefore, random slopes were estimated for case across participant but not for lesion-used or control MRI-used, variables which represent the lesion and control images used to generate the simulated case. This model was tested for both the lesioned hemisphere and contralesional hemisphere, using the *lmer* “subset” argument. When investigating the individual hemispheres, this decreased the number of observations per participant for the random effect of case across participants, and thus a random slope was no longer appropriate. Therefore, only a random intercept was used in the subset analyses.

As hypothesis (b) pertains to the magnitude of differences, the outcome variable was switched to the percent volume difference (PVD) between the simulated-lesion and ground-truth cases. Percentage volume difference for lesioned hemisphere and contralesional hemisphere between simulated-lesion and ground-truth measurements was also calculated as:

$$\begin{array}{l} PVD=100\times \frac{\left|V\left(Simulated\;Lesion\right)-V\left(Ground\;Truth\right)\right|}{\frac{1}{2}\left(V\left(Simulated\;Lesion\right)+V\left(Ground\;Truth\right)\right)} \end{array}$$

where *V* is the volume (calculated for both cortex and cWM volumes), with a greater PVD value representing greater volume differences between the simulated-lesion and ground-truth cases (Perlaki et al., 2017). This is used for two reasons. Firstly, it is a well-accepted approach to segmentation comparison (Fischl et al., 2002) and is used in multiple existing studies of segmentation errors/biases (Morey et al., 2009; Amann et al., 2015; Katuwal et al., 2016; Perlaki et al., 2017). Secondly, it allows us to recode what would be a 2 × 2 interaction (simulated-lesion/ground-truth × lesioned hemisphere/contralesional hemisphere) when using raw volumes as a single factor (PVD of lesioned hemisphere/contralesional hemisphere), meaning that the statistical results reported here are more interpretable. The 20% trimmed means ($\bar{X_{t}}$) and median values for PVD are also reported.

The mixed-effects model was defined similarly to hypothesis (a), but the fixed effect of hemisphere (lesioned/contralesional hemisphere) was included rather than case (simulated-lesion/ground-truth). No random slope of hemisphere across participants was included as there were not sufficient observations per participant to warrant/enable this. Therefore, the maximal model, in this case, included only random intercepts.

As the random effect of control image and lesion significantly improved model fit for both hypothesis (a) and (b), we, therefore, conducted a final, exploratory analyses to investigate how specific characteristics of the lesion, such as lesion size and intensity, explain variance in PVD for both the lesioned hemisphere and contralesional hemisphere. Due to the non-quantitative nature of T1w MR intensity values, voxel intensities of the lesions (in the simulated lesion cases) were calculated in a unit-invariant space, to enable between-participant comparisons of these intensity values. This was achieved by demeaning and scaling intensity values of the MRI of all non-lesioned voxels. These MRI images (where the intensity values were in a unit-invariant space) were only used to calculate lesion characteristics for the exploratory correlation analyses only. These exploratory analyses were conducted using a linear mixed-effects model as per hypothesis (a) and (b), however, the random effect of lesion used was not included, as this variance should be explained by the fixed effects of the lesion characteristics added to the model. The outcome variable was PVD and fixed effects were: lesion size, mean lesion intensity and the SD of lesion intensity, whilst the only random effect was that of control image used to generate the simulated-lesion case. This model was estimated in the lesioned hemisphere and contralesional hemisphere separately, using the *lmer* subset function. Therefore, the random effect of participant was not included as these models no longer represented repeated measures.

For all hypotheses, the mixed-effects model (estimated with maximum likelihood rather than REML to facilitate model comparison) was compared to a linear model including only the fixed effects but none of the random effects to assess whether the random effects were warranted and significantly improved model explanation. All model comparisons were conducted using a Likelihood ratio test to assess whether the reduction in the residual sum of squares was significant. To test the significance of fixed effects concerning all hypotheses, *p*-values were estimated using the normal distribution of *t*-statistics. All results are presented using the “ggplot2” (Wickham, 2009) and “ggpubr” (Kassambara, 2018) packages.

## Results

All models, including parameter estimates for all effects and associated *lmer* syntax, are described in (Supplementary Tables 1–5).

### Differences in Volume Between Simulated Lesion and Ground Truth Cases

For hypothesis (a) both cortex and cWM volume were predicted by the fixed effect of case (simulated-lesion and ground-truth). When adding random effects, for cWM volume, the maximal model failed to converge and thus, as per Barr et al.'s (2013) recommendations, the random correlations between random slope and random intercept were removed from the model, as this performed similarly to the maximal model in simulations (Barr et al., 2013). The addition of the random effects to the model significantly improved model fit for both cortex and cWM volume [χ^2^ (5) = 2,894.47, *p* < 0.0001; χ^2^ (6) = 4,025.22, *p* < 0.0001), and thus the inclusion of random effects in the model was warranted.

For cortex volume, across both hemispheres, the fixed effect of case was non-significant [*B* = −942.61, Standard Error (SE) = 531.83, *t* = −1.77, *p* = 0.076]. When considered separately, surprisingly, the fixed effect of case was non-significant in the lesioned hemisphere (*B* = −1,324.46, SE = 1,155.34, *t* = −1.15*, p* = 0.25), but significant in the contralesional hemisphere (*B* = −560.76, SE = 136.47, *t* = −4.11, *p* < 0.0001). In both hemispheres, the parameter estimates were negative for the simulated lesion case, suggesting that the cortex volume was lower when a lesion was simulated. The significant difference found in the contralesional hemisphere was smaller than the non-significant difference in the lesioned hemisphere. For cWM volume, the effect of case was non-significant across hemispheres (*B* = −161.75, SE = 261.88, *t* = −0.62, *p* = 0.54), and within the individual lesioned hemisphere and contralesional hemisphere, respectively (*B* = −308.84, SE = 619.14, *t* = −0.50, *p* = 0.62; *B* = −14.66, SE = 101.00*, t* = −0.15, *p* = 0.88). These effects can be seen in Supplementary Figure 2.

### Magnitude of Error in Lesioned vs. Contralesional Hemispheres

As hypothesis (b) pertains to the magnitude of differences, the outcome variable was switched to the percent volume difference (PVD) between the simulated-lesion and ground-truth cases. For cortex volume, PVD was slightly higher in the lesioned hemisphere ($\bar{X_{t}}$ = 0.47%, *median* = 0.39%) than the contralesional hemisphere ($\bar{X_{t}}$ = 0.37%, *median* = 0.39%), but overall, the volume difference was minimal between simulated-lesion and ground-truth cases. Only 44 cases showed PVD >1% in the lesioned hemisphere and 27 in the contralesional hemisphere, with maximum PVD being 2.78 and 2.07%, respectively. For cWM volume, PVD was similar between the lesioned hemisphere ($\bar{X_{t}}$ = 0.34%, *median* = 0.31%) and contralesional hemisphere ($\bar{X_{t}}$ = 0.34%, *median* = 0.33%).

For hypothesis (b) the baseline model (including no random effects) was defined similarly as per hypothesis (a). No random slope of hemisphere across participants was utilized as there were not sufficient observations per participant to warrant/enable this. Therefore, the maximal model in this case included only random intercepts and represents a significant improvement over a model with just the fixed effect of hemisphere [cortex volume: χ^2^(3) = 62.19, *p* < 0.0001; cWM volume: χ^2^(3) = 53.71, *p* < 0.0001]. Due to the varying scales across the fixed effect variables, these were converted to z-scores (centered and scaled) to facilitate model convergence.

For PVD of cortex volume, the fixed effect of hemisphere was significant (*B* = 0.140, SE = 0.047, *t* = 2.96, *p* = 0.003), with parameter estimates suggesting that the contralesional hemisphere had a smaller PVD. However, the effect of hemisphere on cWM PVD was non-significant (*B* = −0.007, SE = 0.039, *t* = −0.18, *p* = 0.86). This can be seen in Supplementary Figure 3.

### Lesion Characteristics Associated With Magnitude of PVD

For PVD of both cortex volume and cerebral white matter volume, the random effect of control and lesion used significantly improved model fit. This suggested that there was some variance significantly attributable to the specific lesion used to generate the simulated lesion MRI, as can be seen in Supplementary Figure 4.

Therefore, an exploratory analysis investigated the effects of certain lesion characteristics on PVD. For cortex volume, in the lesioned hemisphere, the fixed effects of lesion volume and SD of lesion intensity were significant (*B* = 0.28, SE = 0.038, *t* = 7.35, *p* < 0.0001; *B* = −0.23, SE = 0.088, *t* = −2.60, *p* = 0.0094, respectively). However, in the contralesional hemisphere, only the fixed effect of SD of lesion intensity was marginally significant (*B* = −0.16, SE = 0.080, *t* = −2.02, *p* = 0.044). For cWM, the only significant fixed effect found was the effect of volume on PVD of cWM in the lesioned hemisphere (*B* = 0.15, SE = 0.030, *t* = 5.08, *p* < 0.0001).

Table 2 summarizes the results pertaining to PVD.

**Table 2A:** Summary of results showing trimmed mean for PVD and fixed effect of hemisphere on these PVD measures.

**Measure**	**Mean PVD**		**Fixed effect of hemisphere**
	**Lesioned hemisphere**	**Contralesional hemisphere**	
Cortex volume	$\bar{X_{t}}$ = 0.47%	$\bar{X_{t}}$ = 0.37%	*B* = 0.140^**^
cWM	$\bar{X_{t}}$ = 0.34%	$\bar{X_{t}}$ = 0.34%	*B* = −0.007

** *p < 0.01*.

**Table 2B:** Summary of effect of lesion characteristics upon PVD measures.

**Measure**	**Lesion characteristic**	**Lesioned hemisphere**	**Contralesional hemisphere**
Cortex volume	Lesion volume	*B* = 0.28^***^	*B* = −0.00
	Mean lesion intensity	*B* = −0.14	*B* = −0.01
	SD lesion intensity	*B* = −0.23^**^	*B* = −0.16^*^
cWM	Lesion volume	*B* = 0.15^***^	*B* = 0.05
	Mean lesion intensity	*B* = 0.03	*B* = 0.02
	SD lesion intensity	*B* = 0.00	*B* = 0.11

* *p < 0.05*;

** *p < 0.01*;

*** *p < 0.001*.

## Discussion

Frank parenchymal lesions as a result of pTBI pathology result in surface reconstruction errors due to abnormal MRI features, such as distortions to the voxel-intensity (Merkley et al., 2008; Irimia et al., 2012; Goh et al., 2014). The current study investigated the accuracy of surface-based, morphometric measurement from T1w images containing TBI-lesions, using a pediatric cohort of simulated lesions and their base control images as a reference. Specifically, we examined whether the lesion-induced error within the Freesurfer pipeline was globally distributed by assessing this error in both the lesion and contralesional hemispheres of the brain.

Statistically significant differences were only found for cortex volume between simulated-lesion and ground-truth cases within the contralesional hemisphere, with the simulated lesions cases having reduced volume. This suggests a significant measurement error introduced to the cortex volume measurement by the lesion, distal to the location of the pathology itself. Surprisingly, no significant differences were found in the lesioned hemisphere for either cortex or cWM volume suggesting that the estimated volumes did not differ when a lesion was simulated. However, this is likely due to the large variance in the effect seen across participants, as shown by the large standard error of the parameter estimates for case in these models for the lesioned hemisphere. This variability is likely because, where pathology is found near to the GM border when the pial and white surfaces are plotted in Freesurfer, these can be erroneously shifted to include WM tissue within the GM ribbon or vice versa (dependant on lesion location). This could, therefore, result in EITHER (a) GM volume reduction or (b) GM volume increase (respectively), hence the high variance in the effect of adding a lesion on measures of brain volume.

It is important to consider that, within the lesioned hemisphere, differences from ground truth segmentation can be thought of as being due to both algorithmic error and “actual” changes: for instance where lesioned tissue is successfully no longer included in the cortical ribbon. However, in the contralesional hemisphere, there has been no image manipulation of the MRI, and thus these reliable, significant differences from the ground truth image are attributable to the lesion-induced error.

Despite the lack of significant differences for cWM volume, descriptive statistics of PVD values, as a measure of the deviation of the simulated-lesion from ground-truth cases, did suggest that there is error, albeit relatively minimal, from the ground truth volumes seen in the cases where a lesion had been simulated. This is seemingly present in both the lesion and contralesional hemispheres and for both cortex volume and cWM. This is in line with our hypothesis of a globally distributed lesion-induced error. However, when comparing PVD between hemispheres to compare the magnitude of these differences, only significant results were found for cortex volume, suggesting that the PVD was greater in the lesioned hemisphere. In terms of the magnitude of differences, we found a maximal PVD from ground truth in the hemispheric cortex and cWM volume of around 2.07–2.78%, respectively. Whilst this represents the extreme cases ($\bar{X_{t}}$ and median values were in the order of around 0.5%), this maximal magnitude of the difference is comparable to that seen for the error induced by minor motion in adult MRI (Reuter et al., 2015).

Overall, the pattern of results we report suggest that whilst the lesion produces large magnitudes of PVD across subjects (PVD was significantly larger in the lesioned hemisphere), this is not consistent across subjects (non-significant differences in cortex volume for the lesioned hemisphere between simulated-lesion and ground-truth) whilst in the contralesional hemisphere, the magnitude of PVD is smaller but is seen consistently across subjects (significant differences in cortex volume for the contralesional hemisphere between simulated-lesion and ground-truth). This holds for cortex, but not cWM volume. These results suggest that, in the contralesional hemisphere, there is a small but systematic bias introduced into the automatic processing and calculation of these morphometric measurements.

We also investigated how MR characteristics of the lesion explained variance in the PVD of our morphometric measures in both the lesioned hemisphere and the contralesional hemisphere. We found that cortex and cWM PVD variance was significantly explained by lesion volume in the lesioned hemisphere, and this was expected as a large lesion will deform the surface to a greater extent, causing greater differences in morphometric measurements. However, more interestingly, we found that in both the lesioned hemisphere and contralesional hemisphere, there was a significant effect of SD of voxel intensities within the lesion on cortex PVD. Specifically, there was a greater difference in cortex volume for lesions with a lower SD of voxel intensities.

One plausible mechanism by which this may be the case is that, where the SD of voxel intensities is high within the lesion, the number of any given “outlier” intensities is low and thus doesn't exceed the noise in the dispersion of voxel intensities across the entire T1w image. However, when the SD is low, there is a higher concentration of potentially “outlier” intensities (especially in lesions of greater volume), and thus these intensities within the lesion may be enough to affect and bias any of Freesurfer's operations which rely on the dispersion of voxel intensities across the image. One example of this may be in the intensity normalization step. Therefore, it is not necessarily the heterogeneous-appearing lesions that would induce the greatest lesion-induced error in both the affected (lesioned hemisphere) and unaffected (contralesional hemisphere) hemispheres, but those lesions which are more homogenous in intensity. However, given the exploratory nature of these correlations, it will be important to perform confirmatory tests on these within an independent pTBI dataset.

Given that Freesurfer processes the two hemispheres separately for a vast proportion of its later pipeline (especially in processing cortical thickness measures), the fact that we see these lesion-induced errors in the hemisphere where there has been no image manipulation of the MRI, suggests that the bias due to lesion-induced error is early in the pipeline. This is in keeping with our findings that the SD of voxel intensities within the lesion effects cortex PVD in both hemispheres, as it is in these early pipeline stages that multiple intensity normalization and correction steps [including an Non-parametric Non-uniform intensity Normalization (N3) correction (Sled et al., 1998)].

The global lesion-induced error was detected in the current study using very coarse measures of brain volume, looking at the entire volume of each hemisphere. It may, in fact, be the case that our estimates of the “error” are conservative overall, and individual ROIs in the contralesional hemisphere may experience a greater error, in differing directions of over- and underestimations. The current investigation precluded ROI analysis of the lesion-induced error across hemispheres due to the fact that the lesioned hemisphere varies between left and right hemispheres for differing lesions. Thus, because many atlases, including those used by Freesurfer do not parcellate the hemispheres with identical homologs, it would be difficult to compare all ROIs between the lesioned hemisphere and contralesional hemisphere. Also, in the presence of gross pathology, probabilistic labeling (such as that performed by Freesurfer to produce ROI volumes) may fail and produce inaccurate registration between the individual and the atlas.

It is important to consider how this may affect previous and future investigations of case/control differences at the group level. Within previous investigations of the pTBI cohort used in the current study, group means for total gray matter differed between pTBI patients and typically developing controls by a PVD value of a similar order of magnitude to the current findings [mild 0.38%, mild-complex 4.8%, moderate 2.7%, and severe TBI 0.77% (Ryan et al., 2016); mild 3.1%, mild-complex 0.93%, moderate 0.66%, and severe TBI 8.1% (Ryan et al., 2017)]. No differences between controls and any TBI severity groups were significant (Ryan et al., 2017). Due to the similar magnitude of changes seen in both real and error-based cases, group-level differences may be contaminated by this error and may erroneously be attributed to pathology-related changes. Whilst this error has been investigated within the context of Freesurfer, it is possible that this error may generalize to other neuroimaging pipeline, especially those that rely upon contrast detection for image processing. Overall, this error has potential ramifications for multiple previous investigations of morphometry.

It is also important to consider the dynamic state of these lesions in the brain. Lesioned tissue within the MRI will likely change in appearance as a function of time, due to effects such as the stabilizing of pathological mechanisms after the acute period, but also potential recovery mechanisms over time. As the lesion changes over time (lesion presentation on MRI is highly dependent on time post-injury (Bigler and Maxwell, 2012), this will result in differences in the lesion-induced error we have detected in the Freesurfer pipeline. These errors may then be misattributed to longitudinal changes to the morphometry of the brain post-pTBI, confounding therapy effects, or “real” recovery measures.

However, it is important to note that not all patients within pTBI studies will present with pathological lesions on T1w MRI and thus the cumulative error from these cases may not exceed the typical “noise” in group-level comparisons. The lesion-induced error, therefore, poses the greatest threat to group-level analysis in those cases where there are small sample sizes, such as can be seen in the existing literature of pathology-related morphometric change to the brain post-pTBI [*n* = 12 (Krawczyk et al., 2010) and *n* = 13 (Urban et al., 2017)], and the relatively few lesion-cases will have a greater artifactual effect on the findings due to limited power to detect true group-differences above and beyond this additional “noise”.

These ideas can be seen in Spanos et al. (2007), who found that group-level reductions in cerebral white matter in a pTBI group compared to typically developing controls were still apparent when excluding those cases where there was a focal lesion. This is an example where, at the group-level, the lesion-induced error we have quantified in this paper seemingly has little effect. We, therefore, recommend that for all group analyses where there are MR lesions present, that a robustness check where lesion-cases are removed and analyses rerun, would be a prudent step to take in assessing the impact of this lesion-induced error on findings. This would be relatively easy to adopt across the field as standard practice, the only difficulty being in those studies where sample sizes are small, and the reduction of statistical power would be too great if these cases were removed.

Of greater concern, however, is the impact of this error on individual-level prediction. Prognostication of cognitive outcome at the individual level is a key goal of many studies investigating TBI, attempting to understand how brain pathology give rise to changes in functional behavior (Bigler, 2016) and therefore aid prediction of long-term outcomes for individuals. However, as noted by Irimia et al. (2012), and subsequently supported by the results of the current study, lesions inappropriately bias the morphometric measurements from automated software packages, thus leading to erroneous measurements of potentially useful biomarkers. In terms of recent methods in medical prognostication using machine learning approaches, this could bias training data in a way that leads to unsuccessful prediction and/or classification of cases. Therefore, the current lesion-induced error renders the subset of pTBI cases which present with pathological lesions on MRI unreachable in terms of prognostication using morphometric measurements of the brain.

Devising a solution to allow for the correction of these individual-level errors in segmentation due to the presence of lesions is non-trivial. Foulon et al. (2017) proposed an approach to study cortical thickness in patients with stroke lesions. They took an approach whereby they enantiomorphically fill the lesion (Nachev et al., 2008) followed by masking cortical thickness within the lesion. Briefly, the enantiomorphic filling is based on the assumption of hemispheric structural symmetry, a chimeric image is produced with the corresponding reflected section of the non-lesioned hemisphere overlaid on the lesioned hemisphere, essentially “filling” the lesion (Nachev et al., 2008). This image is used for calculating the solution to the cost-function in the normalization process, producing a transformation or warp which can later be applied to the non-manipulated T1w image. Thus, the lesion can be transformed without it influencing the spatial normalization process [see Brett et al. (2001) for a further investigation of the effect of lesions on spatial normalization]. Finally, voxels within the lesioned tissue were removed from maps of cortical thickness, thus preventing this “contamination” of measurements. This means that, for an ROI where 50% is covered by lesion tissue, there is still 50% of non-lesioned tissue by which to estimate a mean cortical thickness value for the region. Whilst this was a different software package to Freesurfer, it is important to consider that, within the context of the findings presented here, this approach is unlikely to deal with the global lesion-induced error seen across the brain.

We, therefore, recommend a Freesurfer focal pre-processing approach, similar to the approach by Foulon et al. (2017), whereby the lesioned T1w image is enantiomorphically-filled locally, and this is the image which is processed by Freesurfer. Given our finding that the contralesional hemisphere lesion-induced error seems to be associated with the SD of voxel intensities within the lesion region, it is prudent to think that, by replacing the intensities of this region with homologous intensities from the normal-appearing voxel intensities from within the contralesional regions of the brain, the contralesional lesion-induced error would be mitigated. Therefore, dependent on the quality of the lesion-filling, this would ensure more biologically-meaningful morphometric measurements of the whole-brain to be calculated. As a further Freesurfer post-processing step, individual-subject level atlas parcellations could then be masked as per Foulon et al.'s (2017) approach, whereby region labels which are completely or partially occluded by lesion tissue will be edited. Morphometric measures (such as cortical thickness, volume, etc.) could be calculated using the standard Freesurfer approaches but due to this masking, the output of this pipeline would be cortical morphology measurements which are not contaminated by (a) lesion-tissue within the original image or (b) filled with estimated/imputed voxel intensities in the enantiomorphically filled T1w images. Future studies should investigate the potential of such an approach.

### Limitations

One particular limitation was the drawing of lesion masks. Lesions were identified in our dataset by a single-rater and masks drawn by hand (as is the typical “gold standard” for lesion segmentation of MR images) where visible lesions could be identified by eye using T1w and FLAIR images (by JN). It may, in fact, be the case that some smaller, more subtle lesions were missed and therefore not used in the simulated cases. This, alongside the fact that the array of lesions used as source material for the simulated dataset was small in size (*n* = 16), we cannot ascertain for certain that this effect is ubiquitous to all pTBI cases which present with lesions. However, the purpose of these lesion masks was to allow the extraction of lesion tissue for use in the simulated dataset. Therefore, the issue of false-negatives in the binary voxel masks are less of a concern as lesion tissue was still able to be extracted. Although, it must be acknowledged that potential false-positive identification of lesion voxels in the binary lesion mask is a potential cause for concern, especially as we did not conduct inter- or intra-rater reliability tests.

We aimed to provide specific commentary on the types of lesions observed in TBI, specifically the pathologies seen in our pediatric population. The idea of a “lesion-induced error” to structural segmentation and measurement of morphometry is neither new nor specific to the field of pTBI, and we, therefore, accept that the specific magnitude of error presented here is only generalizable to the current population.

Despite this, the fact that we have utilized “real” lesion cases is beneficial in the sense that it provides us a lesion which retains those characteristics which may be harder to replicate artificially such as texture and the complexity of the distribution of lesions (Seghier et al., 2008). This approach also captures the diverse array of pathology seen post-TBI. Multilevel models presented in the current study showed greater model fit on the inclusion of a random effect of the ID of the lesion used to generate the simulated case. This suggests that the effect of lesions on volume vary across specific lesions. However, the use of these “real” lesions does mean we are limited in the ability to systematically investigate specific lesion characteristics (Chard et al., 2010), such as locale (GM vs. WM vs. Both), size or number (focal vs. multifocal) in comparisons to artificially generated lesions. Figure 1 shows that the spatial distribution of the lesions was in the expected regions [fronto/temporal (Bigler et al., 2016)] but was still varied. There was also a large variation in size of the lesion, focality (multifocal vs. focal), and pathology. This, in turn, makes it difficult to assess which types of lesion are characteristic of this lesion-induced error, or whether the location of the lesion is of specific consequence. Given the current findings that certain characteristics of pathological lesions contribute more/less to the global, lesion-induced error, it may be the case that different pathologies require different correction approaches, dependent on how the pathologies alters the MR signal within the tissue.

## Conclusions

Many previous studies investigating morphometric differences in the brain post-TBI have reported very little information as to how Freesurfer manual-edits have been performed to deal with lesion tissue in some TBI cases. Of those that did, the methods used were post-processing approaches, which dealt with potential error considering only focal errors in the Freesurfer algorithm (Spanos et al., 2007; Drijkoningen et al., 2017). The current study is the first empirical investigation to show that, for cortex volume, in particular, these approaches may not be sufficient. These results suggest that volumetric measures calculated in the presence of lesions are like to show inaccuracies (which are highly variable between individuals) in the lesioned hemisphere, with but also a small, but consistent systematic lesion-induced error being found in the contralesional hemisphere. Thus, this may call into question previous work which has found group differences in brain morphometry, with lesion-induced error being misattributed to pathology-related changes. Future work investigating TBI using morphometric investigations of the brain should be aware of the potential for lesion-induced errors beyond the lesion and be more robust in the reporting of their methods.

## Data Availability Statement

The datasets generated for this study will not be made publicly available Consent was not provided for public sharing at the time of enrolment to the study.

## Ethics Statement

The studies involving human participants were reviewed and approved by Primary study: Royal Children's Hospital, Melbourne. Secondary analysis: Material Transfer Agreement between institutions and ethics & governance agreement for the latter by Aston University. Written informed consent to participate in this study was provided by the participants' legal guardian/next of kin.

## Author Contributions

DK and AW contributed conception and design of the current study. VA contributed and collected data used in the current study. JN contributed lesion masks. DK and AS conceptualized the process of simulating lesions. DK performed the processing of MRI data, performed the statistical analysis, and wrote the first draft of the manuscript. DK and RB conceptualized the statistical analysis. All authors contributed to manuscript revision, read, and approved the submitted version.

## Conflict of Interest

The authors declare that the research was conducted in the absence of any commercial or financial relationships that could be construed as a potential conflict of interest.
