# Supplementary figures and images for: Lesion Induced Error on Automated Measures of Brain Volume: Data From a Pediatric Traumatic Brain Injury Cohort
Source: Front Neurosci. 2020 Nov 30;14:491478. doi: 10.3389/fnins.2020.491478 (PMC7793828; doi:10.3389/fnins.2020.491478)

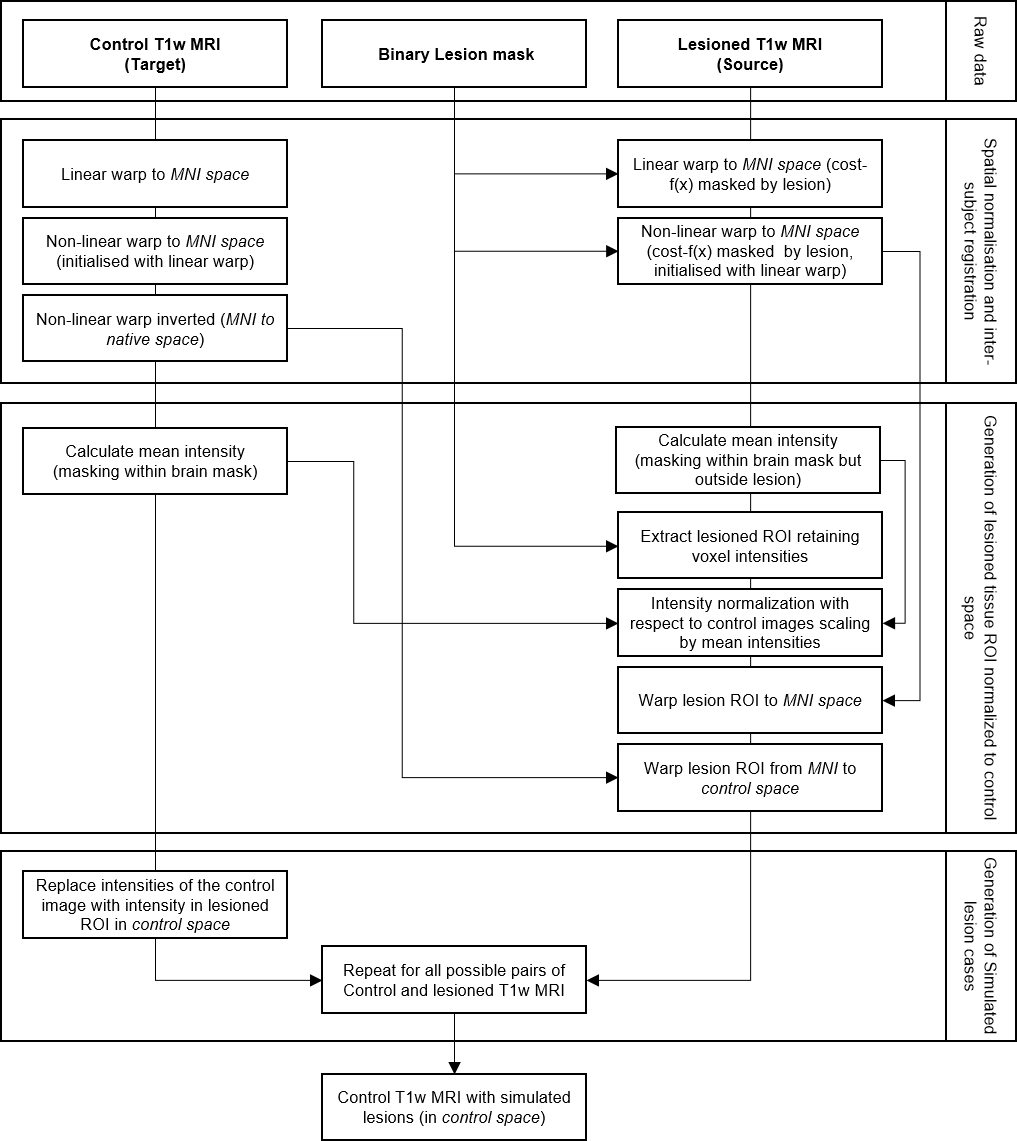

Supplement: Supplementary file 1 [file Figure_1.TIF]

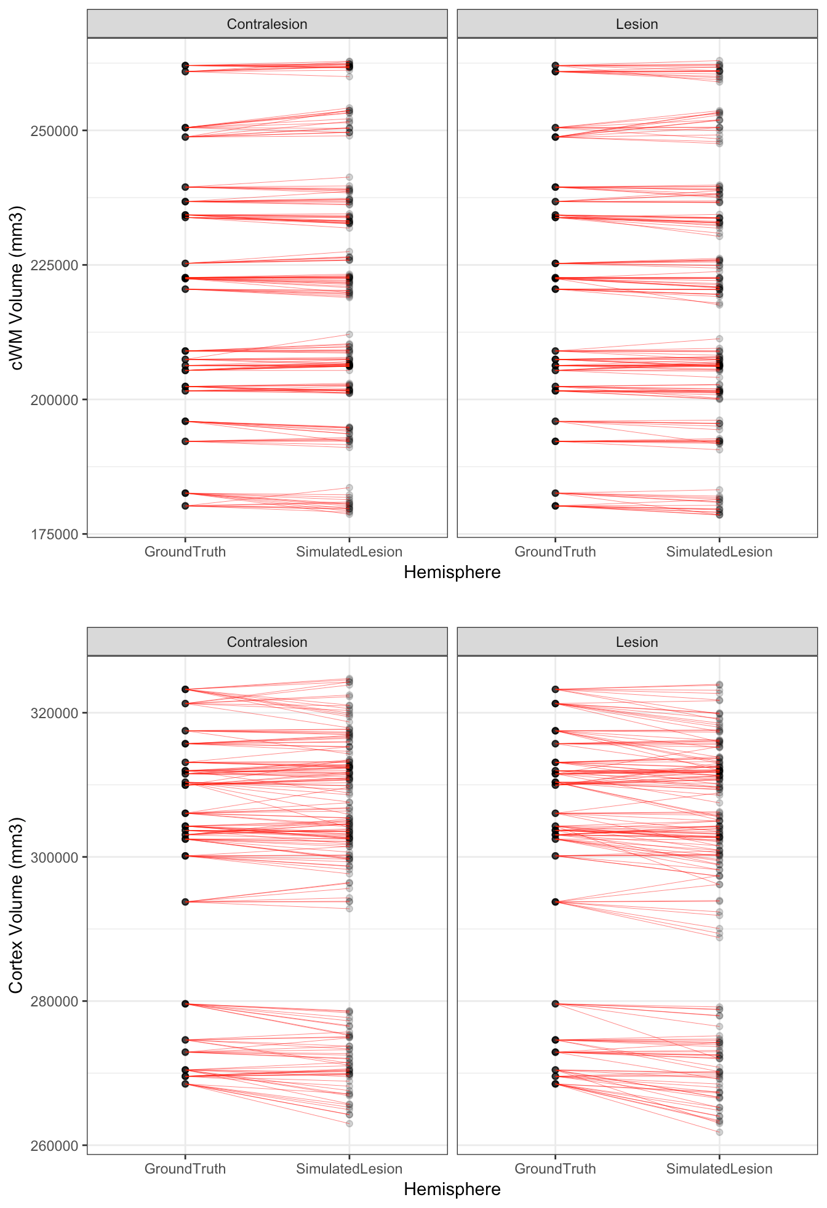

Supplement: Supplementary file 2 [file Figure_2.TIF]

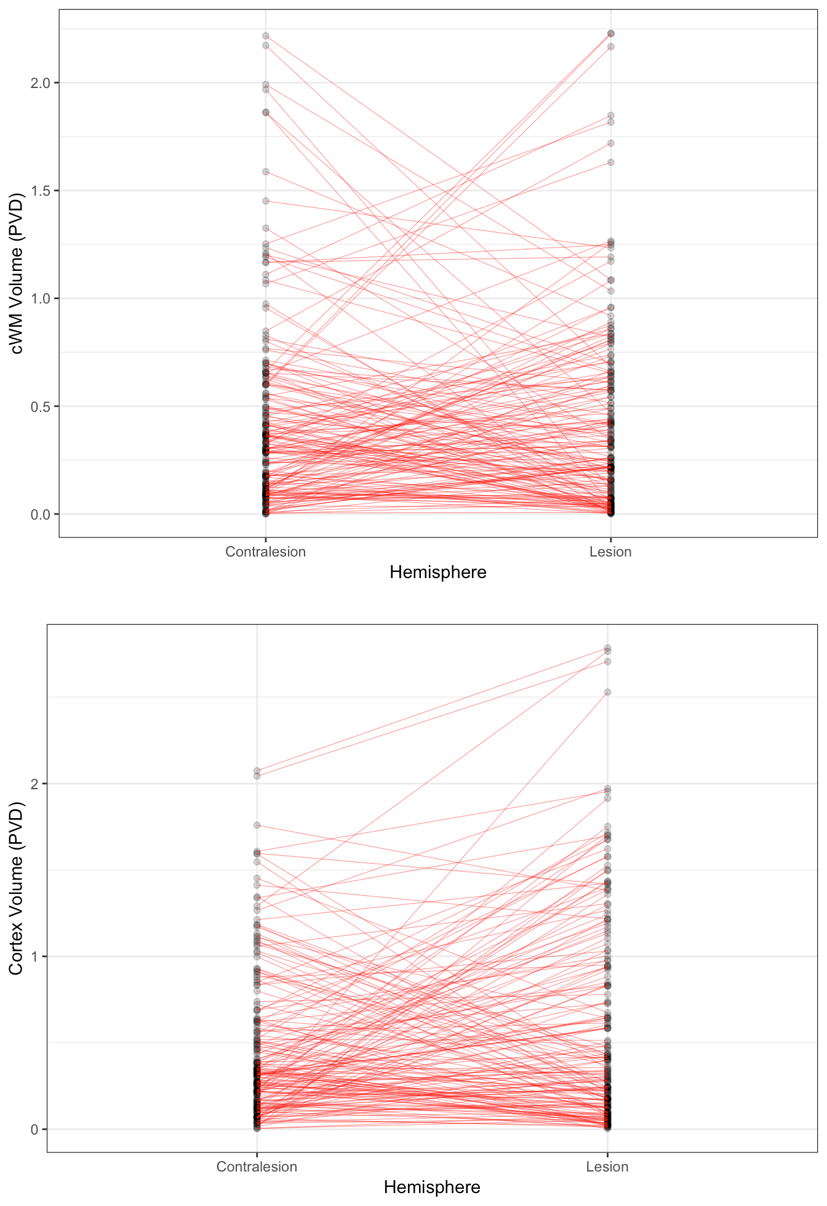

Supplement: Supplementary file 3 [file Figure_3.TIF]

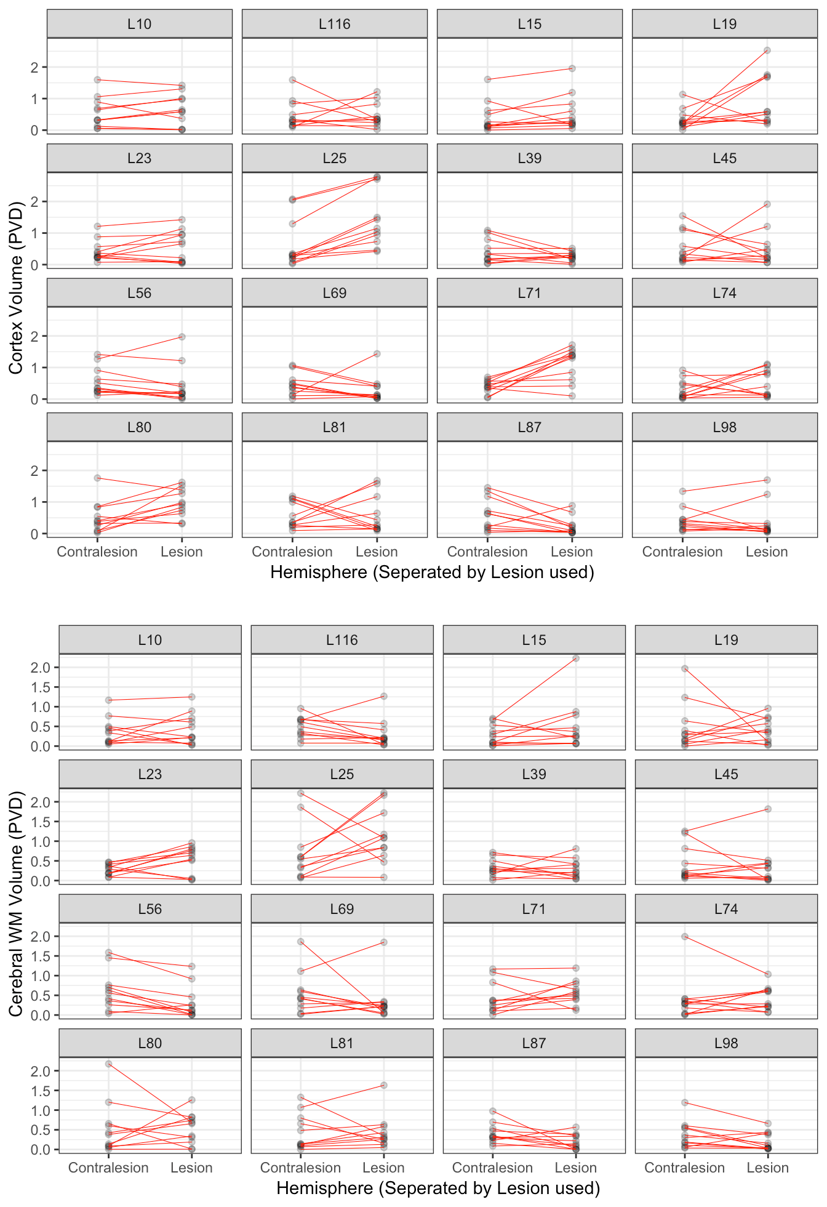

Supplement: Supplementary file 4 [file Figure_4.TIF]
