# Supplementary material for: Lesion Induced Error on Automated Measures of Brain Volume: Data From a Pediatric Traumatic Brain Injury Cohort
Source: Front Neurosci. 2020 Nov 30;14:491478. doi: 10.3389/fnins.2020.491478 (PMC7793828; doi:10.3389/fnins.2020.491478)
Supplement: Supplementary file 5 [file Table_1.DOCX]

Supplementary Material

# Generating simulated lesion cases

Lesions were simulated in control cases from real patient lesions as per previous methods ([Brett, Leff, Rorden, & Ashburner, 2001](#_ENREF_2); [Gonzalez-Villa et al., 2017](#_ENREF_4)). Simulation approaches have been used to investigate the effectiveness of spatial normalization ([Andersen, Rapcsak, & Beeson, 2010](#_ENREF_1); [Brett et al., 2001](#_ENREF_2); [Crinion et al., 2007](#_ENREF_3)) [and segmentation](#_ENREF_19) ([Seghier, Ramlackhansingh, Crinion, Leff, & Price, 2008](#_ENREF_8)) [using focal infarct, vascular event, and atrophic lesions.](#_ENREF_54)

After initially skullstripping all cases (using *FSL*’s ([Jenkinson, Beckmann, Behrens, Woolrich, & Smith, 2012](#_ENREF_6)) [BET](#_ENREF_37) ([Smith, 2002](#_ENREF_9))[), non-linear warps from native space in the skullstripped control and patient cases to MNI-152 space were calculated using](#_ENREF_59) *FSL*’s FNIRT tool (([Jenkinson et al., 2012](#_ENREF_6))[, initialised with an initial 12-DOF linear registration using FLIRT](#_ENREF_37) ([Jenkinson, Bannister, Brady, & Smith, 2002](#_ENREF_5); [Jenkinson & Smith, 2001](#_ENREF_7)) [on the non-skullstripped T](#_ENREF_38)_1_w). For the lesioned cases, this was achieved using cost-function masking, using the inverse of the binary lesion mask (weighting the lesion as zero and the non-lesioned tissue as ones) in order to restrict the warp to being calculated using only healthy tissue. The warp from native control to MNI-152 space was then inverted to produce the warp necessary to move from MNI to control-case native space.

Using the mrcalc function within the *MRTrix* software, the binary lesion mask was multiplied by the lesioned T_1_, resulting in an image that included the lesion tissue only as an ROI. This “patch” was then moved into the native space of every control image, via the MNI-152 space, using the lesioned T1w to MNI-152 warp, followed by the inverse of the control T1w to MNI-152 warp as per below,

$$lesionROI^{targetspace}=lesionROI^{sourcespace}\times warp^{source-to-MNI}\times(warp^{target-to-MNI})^{-1}$$

where the *source* image is the patient T1w and the *target* is the control T1w. To ensure that the lesions are realistic in intensities, we apply a simple intensity normalization procedure between the source and target T1w images. This involved calculation of a scaling factor (SF; as per ([Brett et al., 2001](#_ENREF_2))) as the ratio between the mean intensity of the target image and the source image. This scaling factor was calculated whilst masking the lesion ROI and the abnormal signal intensities within it. This is because different types of pathology will skew mean intensity values (as well as other factors such as size for instance) and thus including lesioned tissue in these calculations would introduce systematic bias into the scaling process. This was then applied to the lesion ROI as follows,

$$lesionROI^{targetintensity}=lesionROI^{sourceintensity}\times\frac{mean^{targetintensity}}{mean^{sourceintensity}}$$

By scaling the lesion ROI in this way, the voxel intensities for the lesion maintain the relative ‘abnormality’ relative to the surrounding healthy tissue in the target image. The scaling factor is applied to the lesion ROI only, so in these simulated cases the voxel intensities of all non-lesioned tissue are identical to if the lesion ROI had not been applied to the image.

Once the lesion ROI has been normalized to the target intensity and space, a binarized and inverted mask of this ROI is generated using mrcalc (where the lesion is zeros and the rest of the image is ones). The target control T1w image is then multiplied by this mask to remove the signal in the target location and then the lesioned ROI in target space and intensity is added to this image (similarly to ([Gonzalez-Villa et al., 2017](#_ENREF_4))) as per below,

$$Simulatedlesion={(lesionmask}^{targetspace}\times T_{1}w^{target})+lesionROI^{{targetspace}/{intensity}}$$

This final image is the simulated lesion case referred to in the rest of the paper.

The resultant simulated dataset contained *n* = 176 cases, where every included lesion (*n* = 16) had been applied to every control image (*n* = 11) in all possible pairwise permutations. From here on, the control images with the simulated lesions applied will be referred to as the simulated lesion (*Sim_lesion_*) cases (*n* = 176) and the control images without editing will be referred to as “ground truth” (*GT*) cases (*n* = 11). The entire pipeline is visualised in the below figure.

[Supp. Figure 1. around here]

**Supplementary Figure 1.** Workflow for generating simulated lesions. Method adapted from ([Brett et al., 2001](#_ENREF_2)) and ([Gonzalez-Villa et al., 2017](#_ENREF_4))

# Mixed Model Constructions

The following table contains descriptions of the models used in the mixed model analyses in *lmer.*

| Supplementary Table 1. Model constructions for mixed model analyses, for each hypothesis. Model designs are constructed using *lmer* syntax | | |
| --- | --- | --- |
| Hypothesis | Subset | Model Construction |
| a) | NA | Outcome ~ Lesionstatus + (Lesionstatus\|Participant) + (1\|Lesion) + (1\|Control_Image) |
|  | Lesioned Hemisphere | Outcome ~ Lesionstatus + (1\|Participant) + (1\|Lesion) + (1\|Control_Image), subset = (Hemi=='Lesion') |
|  | Contralesional Hemisphere | Outcome ~ Lesionstatus + (1\|Participant) + (1\|Lesion) + (1\|Control_Image), subset = (Hemi=='Contralesional') |
| b) | NA | Outcome PVD ~ hemi + (1\|Participant) + (1\|Lesion) + (1\|Control_Image) |
| c) | Lesioned Hemisphere | Outcome PVD ~ Mean_Intensity + SD_Intensity + Volume + (1\|Control_Image) , subset = (hemi=='Lesion') |
|  | Contralesional Hemisphere | Outcome PVD ~ Mean_Intensity + SD_Intensity + Volume + (1\|Control_Image) , subset = (hemi=='Contralesional') |

# Mixed Model Results

| Supplementary Table 2. Outputs from linear mixed-models testing whether the difference in cortex volume between *Sim_lesion_* and *GT* cases is significant across each hemisphere | | | | | | | | | | |
| --- | --- | --- | --- | --- | --- | --- | --- | --- | --- | --- |
|  | | | **Cortex Volume** | | | | | | | |
|  | **Both Hemispheres^a^** | | | | | **Hemi_lesion_^b^** | | | **Hemi_contra_^c^** | |
| *Predictors* | | *B estimates* | | *SE* | *B estimates* | | *SE* | *B estimates* | | *SE* |
| (Intercept) | | 299261.29 ^***^ (288172.71 – 310349.88) | | 5657.55 | 299243.61 ^***^ (287965.38 – 310521.84) | | 5754.30 | 299278.98 ^***^ (288250.11 – 310307.84) | | 5627.08 |
| Simulated Lesion | | -942.61  (-1984.98 – 99.76) | | 531.83 | -1324.46  (-3588.88 – 939.96) | | 1155.34 | -560.76 ^***^ (-828.23 – -293.29) | | 136.47 |
| **Random Effects** | | | | | | | | | | |
| σ^2^ | | | 4544617.35 | | | 1772686.02 | | | 1638835.43 | |
| τ_00_ | | | 0.00 _Participant_ | | | 2938645.92 _Participant_ | | | 2404899.27 _Participant_ | |
|  | | | 241905.00 _Lesion_ | | | 1237325.29 _Lesion_ | | | 0.00 _Lesion_ | |
|  | | | 349283191.51 _Control_Image_ | | | 350327135.17 _Control_Image_ | | | 348051258.85 _Control_Image_ | |
| τ_11_ | | | 0.00 _Participant.LesionstatusSimulatedLesion_ | | |  | | |  | |
| ρ_01_ | | |  | | |  | | |  | |
| ICC | | | 0.00 _Participant_ | | | 0.01 _Participant_ | | | 0.01 _Participant_ | |
|  | | | 0.00 _Lesion_ | | | 0.00 _Lesion_ | | | 0.00 _Lesion_ | |
|  | | | 0.99 _Control_Image_ | | | 0.98 _Control_Image_ | | | 0.99 _Control_Image_ | |
| Observations | | | 704 | | | 352 | | | 352 | |
| Marginal R^2^ /  Conditional R^2^ | | | NA | | | 0.001 / 0.995 | | | NA | |
| *Note. *p<0.05   ** p<0.01   *** p<0.001, ^a^* CortexVol ~ Lesionstatus + (Lesionstatus\|Participant) + (1\|Lesion) + (1\|Control_Image), ^b^ CortexVol ~ Lesionstatus + (1\|Participant) + (1\|Lesion) + (1\|Control_Image), subset = (Hemi=='Lesion'), ^c^ CortexVol ~ Lesionstatus + (1\|Participant) + (1\|Lesion) + (1\|Control_Image), subset = (Hemi=='Contralesional'). | | | | | | | | | | |
|  | | | | | | | | | | |

| Supplementary Table 3. Outputs from linear mixed-models testing whether the difference in cortical white matter volume between *Sim_lesion_* and *GT* cases is significant across each hemisphere | | | | | | |
| --- | --- | --- | --- | --- | --- | --- |
|  | **cWM Volume** | | | | | |
|  | **Both Hemispheres^a^** | | **Hemi_lesion_^b^** | | **Hemi_contra_^c^** | |
| *Predictors* | *B estimates* | *SE* | *B estimates* | *SE* | *B estimates* | *SE* |
| (Intercept) | 220026.04 ^***^ (205476.56 – 234575.53) | 7423.34 | 219983.31 ^***^ (205388.05 – 234578.58) | 7446.70 | 220068.77 ^***^ (205511.02 – 234626.52) | 7427.56 |
| Simulated Lesion | -161.75  (-675.03 – 351.52) | 261.88 | -308.84  (-1522.33 – 904.65) | 619.14 | -14.66  (-212.62 – 183.29) | 101.00 |
| **Random Effects** | | | | | | |
| σ^2^ | 1463696.35 | | 756656.02 | | 897671.31 | |
| τ_00_ | 0.00 _Participant_ | | 721818.41 _Participant_ | | 606868.17 _Participant_ | |
|  | 4521.04 _Participant.1_ | | 352691.35 _Lesion_ | | 0.00 _Lesion_ | |
|  | 55625.95 _Lesion_ | | 606014889.39 _Control_Image_ | | 606761167.60 _Control_Image_ | |
|  | 605508377.50 _Control_Image_ | |  | |  | |
| τ_11_ | 148203.23 _Participant.1.LesionstatusSimulatedLesion_ | |  | |  | |
| ρ_01_ | -1.00 _Participant.1_ | |  | |  | |
| ICC | 0.00 _Participant_ | | 0.00 _Participant_ | | 0.00 _Participant_ | |
|  | 0.00 _Participant.1_ | | 0.00 _Lesion_ | | 0.00 _Lesion_ | |
|  | 0.00 _Lesion_ | | 1.00 _Control_Image_ | | 1.00 _Control_Image_ | |
|  | 1.00 _Control_Image_ | |  | |  | |
| Observations | 704 | | 352 | | 352 | |
| Marginal R^2^ / Conditional R^2^ | NA | | 0.000 / 0.999 | | NA | |
| *Note. *p<0.05   ** p<0.01   *** p<0.001, ^a^* cWMVol ~ Lesionstatus + (1\|Participant) + (0 + Lesionstatus\|Participant) + (1\|Lesion) + (1\|Control_Image), ^b^ cWMVol ~ Lesionstatus + (1\|Participant) + (1\|Lesion) + (1\|Control_Image), subset = (Hemi=='Lesion'), ^c^ cWMVol ~ Lesionstatus + (1\|Participant) + (1\|Lesion) + (1\|Control_Image), subset = (Hemi=='Contralesional'). | | | | | | |
|  | | | | | | |

| Supplementary Table 4. Outputs from linear mixed-models testing whether the magnitude of PVD is significantly different between hemi_lesion_ and hemi_contra_ | | | | |
| --- | --- | --- | --- | --- |
|  | **Cortex PVD ^a^** | | **cWM PVD ^b^** | |
| *Predictors* | *B estimates* | *SE* | *B estimates* | *SE* |
| (Intercept) | 0.48 ^***^ (0.31 – 0.65) | 0.09 | 0.44 ^***^ (0.31 – 0.58) | 0.07 |
| Lesion | 0.14 ^**^ (0.05 – 0.23) | 0.05 | -0.01  (-0.08 – 0.07) | 0.04 |
| **Random Effects** | | | | |
| σ^2^ | 0.20 | | 0.14 | |
| τ_00_ | 0.02 _Participant_ | | 0.02 _Participant_ | |
|  | 0.02 _Lesion_ | | 0.01 _Lesion_ | |
|  | 0.06 _Control_Image_ | | 0.04 _Control_Image_ | |
| ICC | 0.06 _Participant_ | | 0.08 _Participant_ | |
|  | 0.08 _Lesion_ | | 0.06 _Lesion_ | |
|  | 0.19 _Control_Image_ | | 0.18 _Control_Image_ | |
| Observations | 352 | | 352 | |
| Marginal R^2^ / Conditional R^2^ | 0.017 / 0.335 | | 0.000 / 0.319 | |
| *Note. *p<0.05   ** p<0.01   *** p<0.001, ^a^* Cortex PVD ~ hemi + (1\|Participant) + (1\|Lesion) + (1\|Control_Image), ^b^ cWM PVD ~ hemi + (1\|Participant) + (1\|Lesion) + (1\|Control_Image). | | | | |

| Supplementary Table 5. Outputs from linear mixed-models testing whether lesion characteristics can significantly explain variance in PVD across hemi_lesion_ and hemi_contra_ | | | | | | | | |
| --- | --- | --- | --- | --- | --- | --- | --- | --- |
|  | **Cortex PVD** | | | | **cWM PVD** | | | |
|  | **Hemi_lesion_^a^** | | **Hemi_contra_^b^** | | **Hemi_lesion_^c^** | | **Hemi_contra_^d^** | |
| *Predictors* | *B estimates* | *SE* | *B estimates* | *SE* | *B estimates* | *SE* | *B estimates* | *SE* |
| (Intercept) | 0.62 ^***^ (0.48 – 0.76) | 0.07 | 0.48 ^***^ (0.36 – 0.60) | 0.06 | 0.44 ^***^ (0.31 – 0.56) | 0.06 | 0.44 ^***^ (0.31 – 0.57) | 0.07 |
| Mean Intensity | -0.14  (-0.31 – 0.04) | 0.09 | -0.01  (-0.17 – 0.15) | 0.08 | 0.03  (-0.12 – 0.19) | 0.08 | 0.02  (-0.14 – 0.19) | 0.08 |
| SD Intensity | -0.23 ^**^ (-0.40 – -0.06) | 0.09 | -0.16 ^*^ (-0.32 – -0.00) | 0.08 | -0.00  (-0.16 – 0.15) | 0.08 | -0.11  (-0.28 – 0.05) | 0.08 |
| Volume | 0.28 ^***^ (0.20 – 0.35) | 0.04 | -0.00  (-0.06 – 0.06) | 0.03 | 0.15 ^***^ (0.09 – 0.21) | 0.03 | 0.05  (-0.01 – 0.10) | 0.03 |
| **Random Effects** | | | | | | | | |
| σ^2^ | 0.25 | | 0.14 | | 0.15 | | 0.15 | |
| τ_00_ | 0.04 _Control_Image_ | | 0.04 _Control_Image_ | | 0.03 _Control_Image_ | | 0.04 _Control_Image_ | |
| ICC | 0.13 _Control_Image_ | | 0.20 _Control_Image_ | | 0.18 _Control_Image_ | | 0.20 _Control_Image_ | |
| Observations | 176 | | 176 | | 176 | | 176 | |
| Marginal R^2^ / Conditional R^2^ | 0.274 / 0.370 | | 0.121 / 0.294 | | 0.112 / 0.269 | | 0.085 / 0.271 | |
| *Note. *p<0.05   ** p<0.01   *** p<0.001, ^a^* Cortex PVD ~ Mean_Intensity + SD_Intensity + Volume + (1\|Control_Image) , subset = (hemi=='Lesion'), ^b^ Cortex PVD ~ Mean_Intensity + SD_Intensity + Volume + (1\|Control_Image) , subset = (hemi=='Contralesional'), ^c^ cWM PVD ~ Mean_Intensity + SD_Intensity + Volume + (1\|Control_Image) , subset = (hemi=='Lesion'), *^d^* cWM PVD ~ Mean_Intensity + SD_Intensity + Volume + (1\|Control_Image) , subset = (hemi=='Contralesional'), | | | | | | | | |

# Supplementary Figures

[Supp. Figure 2. around here]

Supplementary Figure 2. Plots of differences in cortex (top) and cWM volume (bottom) between simulated-lesion and ground-truth cases across hemispheres.

[Supp. Figure 3. around here]

Supplementary Figure 3. Plots of PVD for both cortex (top) and cWM volume (bottom) across hemispheres for each individual subject.

[Supp. Figure 4. around here]

Supplementary Figure 4. Plots of subject-level PVD for both cortex (top) and cWM volume (bottom) across hemispheres plotted by lesion used.

References

Andersen, S. M., Rapcsak, S. Z., & Beeson, P. M. (2010). Cost function masking during normalization of brains with focal lesions: still a necessity? *Neuroimage, 53*(1), 78-84. doi:10.1016/j.neuroimage.2010.06.003

Brett, M., Leff, A. P., Rorden, C., & Ashburner, J. (2001). Spatial normalization of brain images with focal lesions using cost function masking. *Neuroimage, 14*(2), 486-500. doi:10.1006/nimg.2001.0845

Crinion, J., Ashburner, J., Leff, A., Brett, M., Price, C., & Friston, K. (2007). Spatial normalization of lesioned brains: performance evaluation and impact on fMRI analyses. *Neuroimage, 37*(3), 866-875. doi:10.1016/j.neuroimage.2007.04.065

Gonzalez-Villa, S., Valverde, S., Cabezas, M., Pareto, D., Vilanova, J. C., Ramio-Torrenta, L., . . . Llado, X. (2017). Evaluating the effect of multiple sclerosis lesions on automatic brain structure segmentation. *Neuroimage Clin, 15*, 228-238. doi:10.1016/j.nicl.2017.05.003

Jenkinson, M., Bannister, P., Brady, M., & Smith, S. (2002). Improved optimization for the robust and accurate linear registration and motion correction of brain images. *Neuroimage, 17*(2), 825-841. doi:10.1006/nimg.2002.1132

Jenkinson, M., Beckmann, C. F., Behrens, T. E., Woolrich, M. W., & Smith, S. M. (2012). Fsl. *Neuroimage, 62*(2), 782-790. doi:10.1016/j.neuroimage.2011.09.015

Jenkinson, M., & Smith, S. (2001). A global optimisation method for robust affine registration of brain images. *Medical Image Analysis, 5*(2), 143-156. doi:Doi 10.1016/S1361-8415(01)00036-6

Seghier, M. L., Ramlackhansingh, A., Crinion, J., Leff, A. P., & Price, C. J. (2008). Lesion identification using unified segmentation-normalisation models and fuzzy clustering. *Neuroimage, 41*(4), 1253-1266. doi:10.1016/j.neuroimage.2008.03.028

Smith, S. M. (2002). Fast robust automated brain extraction. *Hum Brain Mapp, 17*(3), 143-155. doi:10.1002/hbm.10062
